# Postoperative Infection and Revision Surgery Rates in Foot and Ankle Surgery Without Routine Prescription of Prophylactic Antibiotics

**DOI:** 10.5435/JAAOSGlobal-D-23-00015

**Published:** 2023-03-08

**Authors:** Neal Huang, Daniel T. Miles, Connor R. Read, Charles C. White, Richard D. Murray, Andrew W. Wilson, Jesse F. Doty

**Affiliations:** From the Department of Orthopaedic Surgery, University of Tennessee College of Medicine Chattanooga, Chattanooga, TN.

## Abstract

Surgical site infections (SSIs) are associated with patient morbidity and increased healthcare costs. Limited literature in foot and ankle surgery provides guidance about routine administration of postoperative antibiotic prophylaxis. The purpose of this study was to examine the incidence and revision surgery rates of SSI in outpatient foot and ankle surgeries in patients not receiving oral postoperative antibiotic prophylaxis.

**Methods:**

A retrospective review of all outpatient surgeries (n = 1517) conducted by a single surgeon in a tertiary referral academic center was conducted through electronic medical records. Incidence of SSI, revision surgery rate, and associated risk factors were determined. The median follow-up was 6 months.

**Results:**

Postoperative infection occurred in 2.9% (n = 44) of the surgeries conducted, with 0.9% of patients (n = 14) requiring return to the operating room. Thirty patients (2.0%) were diagnosed with simple superficial infections, which resolved with local wound care and oral antibiotics. Diabetes (adjusted odds ratio, 2.09; 95% confidence interval, 1.00 to 4.38; *P* = 0.049) and increasing age (adjusted odds ratio, 1.02; 95% confidence interval, 1.00 to 1.04; *P* = 0.016) were significantly associated with postoperative infection.

**Discussion:**

This study demonstrated low postoperative infection and revision surgery rates without the routine prescription of prophylactic postoperative antibiotics. Increasing age and diabetes are signficant risk factors for developing a postoperative infection.

Surgical site infections (SSIs) are associated with increased patient morbidity and increased healthcare costs.^1,2^ The Centers for Disease Control and Prevention has estimated an annual cost of $3.3 billion and a mortality rate of 3% related to SSI.^3^ Specifically, the incidence of foot and ankle SSI in a systematic review was 2% to 4% with rates reported as high as 9%.^4^ Antibiotics are routinely prescribed in the outpatient setting to manage or prevent SSI, although this may not be without some degree of risk. Antibiotic-resistant organisms are an increasing and evolving problem when trying to treat clinical infections. Recent Centers for Disease Control and Prevention estimates suggest that two million annual antibiotic-resistant infections lead up to 23,000 yearly deaths.^5^ Physicians and healthcare systems must be cognizant of their routine prescribing practices and the possible implications of such. Many healthcare institutions now have initiatives to decrease antimicrobial resistance by developing antibiotic stewardship programs. The development of structured guidelines and clinical pathways further promotes the safest prescribing practices.

For decades, general surgery and trauma publications were referenced to suggest the routine efficacy of perioperative surgical antibiotic prophylaxis in preventing SSI in major operations.^6-8^ Ruta et al^9^ conducted a survey of foot and ankle surgeons regarding postoperative antibiotics for outpatient surgeries. Seventy-five percent of responding surgeons reported the use of oral postoperative antibiotic prophylaxis in some patients while 16% of respondents prescribed an oral postoperative antibiotic to all patients. A paucity of literature regarding the most efficacious utility of antibiotic prophylaxis in foot and ankle surgery leaves surgeons without clear guidance on antibiotic administration. With the growing pressure of antimicrobial stewardship in medicine, it is necessary to establish whether current prescribing patterns are effective or necessary in limiting infections. The purpose of this study was to determine the incidence of SSI and subsequent effect on revision surgery rates in patients not receiving oral postoperative antibiotic prophylaxis. Secondary identification of high-risk comorbidities that predispose patients to getting an SSI may warrant a lower threshold for antibiotic prophylaxis. The authors have hypothesized that outpatient surgery without routine postoperative antibiotic prophylaxis leads to acceptable surgical outcomes.

## Methods

### Study Population

A retrospective cohort study was approved by the university scientific review committee and the institutional review board at the hospital system where all procedures were conducted. An electronic health record query identified 1685 patients who underwent outpatient elective surgery with a single fellowship-trained orthopaedic foot and ankle surgeon over a four-year period from January 1, 2017, to May 1, 2020. Inclusion criteria consisted of patients of all ages, and any Current Procedural Terminology code, undergoing same-day discharge surgical procedures. Patients who were admitted to the hospital postoperatively for 23-hour observation, inpatient stays, and those who underwent a surgical procedure for a preexisting infection were excluded. All patients received preoperative antibiotics of cefazolin, and if they were allergic to cephalosporins, vancomycin was to be given. Per surgeon preference, no patients received any immediate postoperative antibiotics. In total, 1517 patients met the inclusion criteria (Table 1). The median age was 47 years (interquartile range [IQR], 32 to 59 years). The median body mass index (BMI) was 29.6 kg/m^2^ (IQR, 25.2 to 34.8 kg/m^2^). Male patients represented 34.1% of the cohort, and female patients represented 65.9%. The median follow-up was 6 months (IQR, 3 to 9 months).

**Table 1:** Characteristics of Patients Who Underwent Outpatient Elective Surgery

Characteristic	Total (n = 1517)	Infection		
		Overall (n = 44)	Superficial (n = 30)	Deep (n = 14)
Age				
Median (IQR)	47 (32 to 59)	52 (45 to 61)	57 (46 to 61)	46 (41 to 61)
<20	155 (10.2%)	1 (2.3%)	1 (3.3%)	0 (0.0%)
20-39	396 (26.1%)	5 (11.4%)	2 (6.7%)	3 (21.4%)
40-59	615 (40.5%)	23 (52.3%)	16 (53.3%)	7 (50.0%)
≥60	351 (23.1%)	15 (34.1%)	11 (36.7%)	4 (28.6%)
Sex				
Male	518 (34.1%)	20 (45.5%)	14 (46.7%)	6 (42.6%)
Female	999 (65.9%)	24 (54.5%)	16 (53.3%)	8 (57.1%)
Follow-up (mo)	6 (3 to 9)	4.5 (3 to 10)	4 (3 to 7.2)	5.5 (2.7 to 12.2)
BMI				
Median (IQR)	29.6 (25.2 to 34.8)	29.6 (25.2 to 34.8)	32.6 (26.5 to 40.4)	31.4 (25.3 to 35.7)
<25	347 (22.9%)	8 (18.2%)	5 (16.7%)	3 (21.4%)
25-29	436 (28.7%)	11 (25.0%)	8 (26.7%)	3 (21.4%)
30-39	566 (37.3%)	15 (34.1%)	9 (30.0%)	6 (42.9%)
≥40	168 (11.1%)	10 (22.7%)	8 (26.7%)	2 (14.3%)
Preoperative antibiotics				
Cefazolin	1428 (94.1%)	43 (97.7%)	29 (96.7%)	14 (100.0%)
Clindamycin	2 (0.1%)	0 (0.0%)	0 (0.0%)	0 (0.0%)
Vancomycin	87 (5.8%)	1 (2.3%)	1 (3.3%)	0 (0.0%)
Diabetes	170 (11.2%)	11 (25.0%)	9 (30.0%)	2 (14.3%)
Tobacco use	466 (30.7%)	18 (40.9%)	12 (40.0%)	6 (42.9%)
Hypertension	468 (30.9%)	19 (43.2%)	12 (40.0%)	7 (50.0%)
COPD	20 (1.3%)	1 (2.3%)	1 (3.3%)	0 (0.0%)
Cardiovascular disease	47 (3.1%)	2 (4.5%)	2 (6.7%)	0 (0.0%)
Stroke	16 (1.1%)	1 (2.3%)	0 (0.0%)	1 (7.1%)
Cancer	59 (3.9%)	1 (2.3%)	0 (0.0%)	1 (7.1%)
Hypothyroidism	92 (6.1%)	5 (11.4%)	4 (13.3%)	1 (7.1%)
Thyroid disease	58 (3.8%)	0 (0.0%)	0 (0.0%)	0 (0.0%)
Rheumatoid arthritis	41 (2.7%)	2 (4.5%)	1 (3.3%)	1 (7.1%)
Gout	28 (1.8%)	3 (6.8%)	3 (10.0%)	0 (0.0%)
Anemia	24 (1.6%)	2 (4.5%)	1 (3.3%)	1 (7.1%)
Neuropathy	21 (1.4%)	2 (4.5%)	2 (6.7%)	0 (0.0%)
DVT history	16 (1.1%)	0 (0.0%)	0 (0.0%)	0 (0.0%)
MRSA history	11 (0.7%)	0 (0.0%)	0 (0.0%)	0 (0.0%)
Hyperthyroidism	7 (0.5%)	0 (0.0%)	0 (0.0%)	0 (0.0%)

BMI = body mass index, COPD = chronic obstructive pulmonary disease, DVT = deep vein thrombosis, IQR = interquartile range, MRSA = methicillin-resistant *Staphylococcus aureus*

Data represented as number (%) or median (IQR).

### Measured Outcomes

The primary outcome measured was SSI rate and the subsequent need for revision surgery because of infection. Parameters reviewed included age, sex, BMI, tobacco use, medical comorbidities, procedure type, length of postoperative follow-up, need for wound care, and revision surgery rates. Patients who were subsequently prescribed antibiotics, because of wound healing concerns, at the time of outpatient follow-up, were further subcategorized as prophylactic antibiotics, superficial infections, and deep infections.

### Definition of Infection

Superficial infections were defined by erythema, wound drainage, suture abscesses, and excessive warmth at the surgical site. Superficial wound dehiscence without any clinical signs of infection were not counted as infected, and if antibiotics were given, these patients were categorized as prophylactic antibiotics. Deep infections were delineated as those who required a return to the operating room for irrigation and débridement.

### Statistical Analysis

Statistical analyses were conducted with IBM SPSS Statistics version 27.0 (IBM). Tests were conducted 2-tailed and a *P* < 0.05 defined statistical significance. Patient characteristics are expressed as frequencies (%) and median (IQR). Normality was assessed by using the Shapiro-Wilk test (*P* > 0.05) and Q-Q plot. Chi-square test, Fisher's exact test, and univariate logistic regression were used to determine signficant associations between infection and risk factors. To adjust for confounding, multivariate logistic regression was used with clinically and statistically significant variables. Linearity of continuous variables was assessed with the Box-Tidwell procedure. Model fit was assessed with the Hosmer-Lemeshow test (*P* > 0.05). Multicollinearity was absent, as assessed by tolerance values greater than 0.1. Fewer than five and one percent of the standardized residuals laid outside two and three standard deviations. Cook's distance values more than 1.0 were absent.

## Results

Forty-four patients (2.9%) experienced postoperative infection, and 14 patients (0.9%) required returning to the operating room because of infection. Of these 14 cases, 10 index surgeries involved the ankle and hindfoot. These procedures consisted of two ankle fractures, one pilon fracture, one talus fracture, two Achilles repairs, two peroneal tendon repairs with lateral ligament repairs, one tarsal coalition excision, and one anterior tibialis tendon repair. The remaining four cases were forefoot deformity corrections. The median time between index surgery and revision surgery was 77 days (IQR, 39 to 169 days). Eleven of the 14 revision surgery patients (78.6%) were older than 40 years. Eight patients (57.2%) had a BMI greater than 30 kg/m^2^. Seven patients (50.0%) had hypertension, and six (42.9%) used tobacco.

Thirty patients (2.0%) were diagnosed as simple superficial infections that resolved with local wound care. Treatment also consisted of a prescription of Bactrim 800/160 mg two times per day for 7 to 10 days (27 of 30) or clindamycin 300 mg three times per day for 7 days (2 of 30). One superficial infection resolved without antibiotics. Twenty-seven of the superficial patients (90.0%) were older than 40 years. Seventeen patients (56.7%) had a BMI greater than 30 kg/m^2^. Twelve patients (40.0%) had hypertension and used tobacco. Diabetes was the most signficant risk factor and accounted for 30.0% (9 of 30) of the superficial infections (adjusted odds ratio [OR], 2.53; 95% confidence interval [CI], 1.09 to 5.87; *P* = 0.031).

Overall, patients older than 40 years accounted for 86.4% of the postoperative infections (38 of 44), highlighting increasing age as a significant predictor (adjusted OR, 1.02; 95% CI, 1.00 to 1.04; *P* = 0.016). Eleven of the 44 patients (25.0%) had diabetes, which was the most signficant risk factor in both univariate and multivariate analyses (adjusted OR, 2.09; 95% CI, 1.00 to 4.38; *P* = 0.049) (Tables 2 and 3). Ten patients (22.7%) had a BMI greater than 40 kg/m^2^. Cefazolin was the most frequently used preoperative antibiotic (97.7%). Eighteen patients (1.1%) developed superficial wound dehiscence without clinical signs of infection, of whom 10 patients received prophylactic antibiotics for treatment.

**Table 2:** Univariate Analysis Between Postoperative Infection and Risk Factors

Risk Factor	Infection (n = 44)		Superficial (n = 30)		Deep (n = 14)	
	OR (95% CI)	*P* Value	OR (95% CI)	*P* Value	OR (95% CI)	*P* Value
Diabetes	2.75 (1.36 to 5.55)	0.007^a^	3.53 (1.58 to 7.83)	0.004^a^	1.32 (0.29 to 5.96)	0.665
Age (per yr)	1.02 (1.00 to 1.04)	0.020^a^	1.03 (1.00 to 1.05)	0.008^a^	1.01 (0.98 to 1.04)	0.404
BMI (per kg/m^2^)	1.02 (0.98 to 1.06)	0.227	1.04 (0.99 to 1.08)	0.075	1.01 (0.94 to 1.08)	0.769
Sex (male)	1.63 (0.89 to 2.98)	0.145	1.70 (0.82 to 3.52)	0.173	1.45 (0.50 to 4.20)	0.573
Tobacco use	1.58 (0.86 to 2.91)	0.183	1.51 (0.72 to 3.17)	0.317	1.70 (0.58 to 4.92)	0.383
Hypertension	1.73 (0.94 to 3.18)	0.096	1.50 (0.72 to 3.15)	0.318	2.26 (0.78 to 6.48)	0.146

BMI = body mass index, CI = confidence interval, OR = odds ratio

a Statistically significant (*P* < 0.05).

**Table 3:** Multivariate Analysis Between Postoperative Infection and Risk Factors

Risk Factor	Infection (n = 44)		Superficial (n = 30)		Deep (n = 14)	
	OR (95% CI)	*P* Value	OR (95% CI)	*P* Value	OR (95% CI)	*P* Value
Diabetes	2.09 (1.00 to 4.38)	0.049^a^	2.53 (1.09 to 5.87)	0.031^a^	1.15 (0.24 to 5.47)	0.860
Age (per yr)	1.02 (1.00 to 1.04)	0.016^a^	1.03 (1.00 to 1.05)	0.019^a^	1.01 (0.98 to 1.04)	0.407
BMI (per kg/m^2^)	1.02 (0.98 to 1.07)	0.199	1.03 (0.98 to 1.08)	0.164	1.01 (0.93 to 1.08)	0.795
Sex (male)	1.79 (0.97 to 3.29)	0.061	1.90 (0.91 to 3.96)	0.084	1.51 (0.52 to 4.40)	0.446

BMI = body mass index, CI = confidence interval, OR = odds ratio

a Statistically significant (*P* < 0.05).

## Discussion

Based on historical data, systemic perioperative antibiotic prophylaxis has been routinely prescribed for orthopaedic procedures for decades. This study demonstrated low infection and revision surgery rates after elective outpatient foot and ankle surgery without the routine use of postoperative prophylactic antibiotics. Increasing age and diabetes were signficant risk factors of postoperative infection. No other patient demographic or comorbidity showed an increased risk of superficial infection or deep infection requiring débridement in the operating room. We report an infection rate of 2.9% and a revision surgery rate for infection of 0.9%, which are within the previously reported parameters (range, 3.0% to 4.8%) using postoperative antibiotic prophylaxis.^10-12^ There are limited studies in foot and ankle research for the use of routine prophylactic postoperative antibiotics. Frederick et al^13^ reported a 2.3% infection rate with 0.8% deep infection rate in 1227 patients who underwent foot and ankle surgery and did not receive postoperative oral antibiotics. No significant difference was found when this group was compared with a postoperative antibiotic prescription group with a 3.4% infection rate (*P* = 0.08). Our results support these findings.

Multiple modifiable and nonmodifiable patient comorbidities have been suggested to increase the risk of postoperative infection.^10-12^ Diabetes with increased hemoglobin A1c preoperatively is a modifiable risk factor that has a direct correlation with postoperative infection.^10,14-16^ Patients with diabetes were at an increased risk of developing a postoperative infection (OR, 2.75, *P* = 0.007) in a univariate analysis and were at an increased risk of superficial infection but failed to be associated with return to the operating room. This lack of significance is likely due to the low number of cases requiring revision surgery. Multivariate regression analysis demonstrated increased risk (adjusted OR, 2.09; *P* = 0.049) in developing postoperative infections when adjusting for age, BMI, and sex.

Obesity is another risk factor that has been implicated in postoperative infection and wound complications across orthopaedics.^17^ This study did find that 56.8% of the infection patients had a BMI of >30 kg/m^2^ and 22.7% had a BMI of >40 kg/m^2^. Olsen et al^18^ found having a BMI of >30 kg/m^2^ to be a signficant predictor of both superficial and deep infections (OR, 1.68) in the treatment of ankle fractures. To our knowledge, this is the first study to show the detrimental effect that obesity, independent of diabetes, can have on patients undergoing elective foot and ankle surgery.

The implication of increasing age on postoperative infection has been heavily disputed. The effect of age on infection is important to examine because it is a nonmodifiable risk factor. Multiple studies have shown associations with increasing age and postoperative infection.^19,20^ This suggests a positive role for prophylactic antibiotic considerations in the elderly. However, this does not come without signficant risk as a population disproportionately affected by opportunistic infections from antibiotics.^21^ Our study demonstrated an age-related propensity for postoperative superficial infections that were treated with antibiotics and subsequently resolved without additional sequelae. Patients who were 40 years or older accounted for 86.4% of the postoperative infections. Carl et al^11^ found a similar signficant association with increasing age when comparing the average age of the infection group (age 55 years) with that of the no-infection group (age 45 years). Wiewiorski et al,^20^ in a prospective study, demonstrated age 60 years and older to be an independent risk factor of wound complications in an elective foot and ankle population. Age as a causative factor of infection is difficult to determine because older individuals typically have more comorbidities. However, increasing age was significant in developing a superficial infection after correcting for confounding comorbidities (adjusted OR, 1.03 per year; *P* = 0.019). This suggests that it would be beneficial for prophylactic antibiotics to be prescribed in an older population because they are at higher risk of superficial infections. Our results add to the growing body of literature that age likely plays a role in developing infection postoperatively.

This study is limited by its retrospective design and lack of a true control group. Historical infection rates were used as a control group, but population demographics may be different. This study also depended heavily on electronic medical records with the presumption of accurate patient data and follow-up infection documentation. Therefore, there was no standardization of antibiotic prescription practice that may leave room for subjective judgment. Multivariate logistic regression analysis is also susceptible to bias because it cannot adjust for all unidentified risk factors that could be clinically relevant. In addition, quantification of diabetes was not conducted. To the authors’ knowledge, this is the largest population on the topic to date and the study's main strength. An additional strength is the variety of procedures included in this study represent those typically experienced by foot and ankle surgeons.

This study demonstrated low postoperative infection and revision surgery rates without prescribing prophylactic postoperative antibiotics in outpatient foot and ankle surgery patients. We identified increasing age and diabetes as signficant risk factors for developing a postoperative infection. No other patient demographic or comorbidity showed an increased risk of superficial infection or deep infection. These findings suggest that outpatient foot and ankle surgery without routine postoperative antibiotic prophylaxis leads to an acceptable postoperative SSI incidence.
